# Paleolithic Murals and the Global Wildlife Trade

**DOI:** 10.3201/eid1107-AC1107

**Published:** 2005-07

**Authors:** Polyxeni Potter

**Affiliations:** * Centers for Disease Control and Prevention, Atlanta, Georgia, USA

**Keywords:** Paleolithic period, stone age, prehistory, cave painting, Lascaux, wildlife trade

**Figure:**
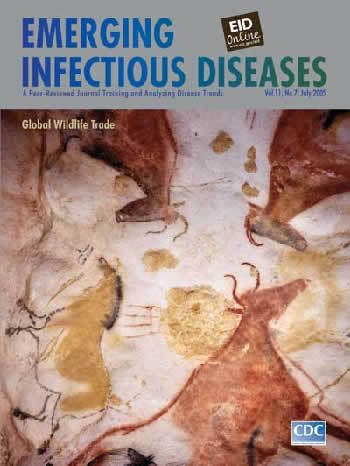
**The Painted Gallery (17,000 BC).** Ceiling, Lascaux Caves, Perigord, Dordogne, France. Image courtesy of Ministry of Culture and Communication, National Centre of Prehistory, France

"Antiquities are history defaced," argued Renaissance philosopher Francis Bacon in The Advancement of Learning; they are "…remnants of history which…escaped the shipwreck of time" (*1*). These remnants, battered by the elements and scattered around the globe, are all we have to piece together human heritage. In recent years, radiocarbon dating techniques (e.g., accelerator mass spectrometry) have allowed us to date samples of pigment from cave paintings and not rely solely on evidence from surrounding artifacts. These techniques shed light on the chronology and evolution of prehistoric art and show that cave painting began much earlier than believed, as early as the Upper Paleolithic period (*2*).

The Paleolithic period (Old Stone Age), the earliest stretch of human history 2 million years ago, saw the development of the human species. Nomadic hunters and gatherers, who lived in caves and crafted tools out of stone, progressed during the last ice age to communal hunting, constructed shelters, and belief systems. The Upper Paleolithic (end of Old Stone Age) marks humanity's cognitive and cultural, as well as artistic, beginnings (*3*).

When, why, and how precisely humans moved from rote tool-making to symbolic self-expression is not known. Evidence gathered in the past 200 years indicates that graphic activity (figurative and nonfigurative marks) began as early as 40,000 years ago, preceding the development of agriculture. It coincided with human migration around the globe, the production of implements from multiple materials, and the building of simple machines. Images carved, etched, or painted on stone, clay, bone, horn, ivory, or antler were found in Africa, Australia, the Middle East, and Europe, and portable sculptures of animal and human figures were more common and widespread than cave paintings (*4*).

The first discoveries of Paleolithic painting (40,000 BC–12,000 BC), which were greeted with skepticism and disbelief, were made around 1835 in France and Switzerland (*5*) and later in Spain, Australia, South Africa, and other places. The western edges of the Massif Central region of France and the northern slopes of the Pyrenees are dotted with more than a hundred Paleolithic caves, among them renowned Lascaux. Naturally blocked for thousands of years, these deep caves maintained sufficiently stable temperature and humidity to preserve, untouched, not only paintings but also footprints and handprints of their inhabitants. After the discovery of Lascaux in 1940, environmental conditions changed. A fungus (*Fusarium*) began to grow inside the cave, and algae spread on the floor, walls, and ceiling, threatening the integrity of the paintings. The threat was contained, but access to the public was curtailed (*6*).

The cavern "gave onto a steep slope, slippery and slimy…with flakes of worked flint of poor quality, some fragments of reindeer horns and many pieces of conifer charcoal…" recalled speleologist Abbé Breuil about the initial opening to Lascaux (*7*). The ground was different 17,000 years ago. The cave had sunk and was difficult to reach, the entrance obscured by millennia of erosion and sediment.

The dark sanctuary contained a network of passages, caverns, and shafts, later named Great Hall of the Bulls, Painted Gallery, Chamber of Engravings, Chamber of Felines, Shaft of the Dead Man (*8*). Uneven wall and ceiling surfaces sported enigmatic scratches, smudges, combinations of dots and grids, as well as drawings and painted figures whose narrative meaning had been rendered unfathomable by the passage of time.

Mineral pigments (ochre, charcoal, iron oxide, hematite, manganese) were ground into animal fat and applied with brushes or were blown through hollow sticks. The artists, working in confined, possibly dangerous space, under unsteady or flickering light from a torch or oil lamp, incorporated distortions and shadows in the design, along with soot.

As if suddenly awakened from extended hibernation, herds of aurochs, horses, chamois, ibexes, bison, stags, rhinoceroses rush en masse on the cave walls. Large wild beasts, drawn not from life but from the imagination, populate free-flowing compositions in coordinated movement with each other. Their bodies, intently outlined and punctuated with color, at once realistic and stylized, conform to the contours of the cave, the cracks and imperfections of which are often incorporated in the drawing. Anatomical details betray familiarity with the animals, as well as observational and artistic skill. Proportions are mostly accurate, except for the heads, which tend to be small, and the horns, which are sometimes exaggerated. Unrestricted and unbound, the beasts frolic on the dark walls, at times overlapping as they gallop toward or away from each other.

A strong "occluding contour," an essential silhouette, is etched or painted on the hard surfaces. The silhouette, often the only graphic, boasts a prominent cervicodorsal line, in profile. And this line is at times the only line, as if part of the animal is drawn to suggest the whole. Even though figures are presented in profile, distinctive details (horns or hooves) have an independent, "twisted" orientation (*9*).

Large animals are the protagonists. Humans, if present at all, are stick figures, masked or headless, crudely drawn, stiff and nonexpressive, their puzzling presence possibly symbolic and secondary. We do not know if these murals represent early social interactions (the hunt, sacred rites, tribal ceremonies); hallucinogenic imagination inflamed by fumes in unventilated caves; unknown primitive rituals; or simply artistic compulsion. Yet, this preferential and exuberant treatment of animals suggests on the part of our ancestors inexplicable fascination with wildlife.

As far back as the Paleolithic age, humans have lived in close proximity with animals, associating not only with those they could domesticate but also with wild and dangerous beasts. Encounters contained an element of risk, for humans were injured or killed as much as nourished or entertained. The enigmatic portrayal of large, wild beasts on the walls and ceiling at Lascaux suggests a complex early relationship that went beyond the necessities of food or fiber. In our time, interaction with animals continues to encompass cohabitation at all levels, including the microbial. Encounters, compounded by increased travel and trade, still involve risks as well as benefits. And even though we are less likely to be injured or killed by animals, the exotic pathogens living and traveling with them counterbalance amusement and companionship with illness and death (*10*).
